# Neuroprotective Effects of Salidroside on Cerebral Ischemia/Reperfusion-Induced Behavioral Impairment Involves the Dopaminergic System

**DOI:** 10.3389/fphar.2019.01433

**Published:** 2019-12-13

**Authors:** Zhi-feng Zhong, Jing Han, Ji-Zhou Zhang, Qing Xiao, Jing-yan Chen, Kai Zhang, Juan Hu, Li-dian Chen

**Affiliations:** ^1^Institute of Materia Medica, Fujian Academy of Traditional Chinese Medicine, Fuzhou, China; ^2^Department of High Altitude Operational Medicine, College of High Altitude Military Medicine, Army Medical University (Third Military Medical University), Chongqing, China; ^3^Fujian Key Laboratory of Natural Medicine Pharmacology, Fujian Medical University, Fuzhou, China; ^4^School of Rehabilitation Medicine, Fujian University of Traditional Chinese Medicine, Fuzhou, China

**Keywords:** salidroside, neuroprotection, cerebral ischemia/reperfusion, dopaminergic system, microdialysis

## Abstract

Salidroside, a phenylpropanoid glycoside, is the main bioactive component of *Rhodiola rosea* L. Salidroside has prominent anti-stroke effects in cerebral ischemia/reperfusion models. However, the underlying mechanisms of its actions are poorly understood. This study examined the anti-stroke effects of salidroside in middle cerebral artery occlusion (MCAO)-induced rat model of stroke and its potential mechanisms involving the dopaminergic system. Salidroside administration increased the levels of dopamine (DA), homovanillic acid (HVA), and 3,4-dihydroxyphenylacetic acid (DOPAC) in the ipsilateral striatum after induction of transient ischemia, which were assessed using microdialysis with high-performance liquid chromatography coupled with electrochemical detection (HPLC-ECD). Furthermore, treatment with salidroside ameliorated neurobehavioral impairment, assessed with the modified neurological severity scores (mNSS), the balance beam test, and the foot fault test. Moreover, enzyme-linked immunosorbent assay (ELISA) suggested that MCAO-induced reduction in monoamine oxidase (MAO) was inhibited by salidroside. Immunohistochemical and immunofluorescence analyses revealed high level of tyrosine hydroxylase (TH) in the ipsilateral striatal caudate putamen (CPu) after cerebral ischemia/reperfusion, which could be further elevated by salidroside. In addition, salidroside could reverse the decreased immunoreactivity of TH in the substantia nigra pars compacta (SNpc). These results suggest that the anti-stroke effects of salidroside in MCAO-induced cerebral ischemia/reperfusion may involve the modulation of monoamine metabolism in the striatum and SNpc, which may be related to the function of the dopaminergic system in the rat brain.

## Introduction

According to the World Health Organization, stroke is the leading cause of death and permanent disability in adults worldwide (Benjamin et al., 2017; Benjamin et al., 2018). Approximately 87% of strokes are ischemic (Benjamin et al., 2018), and they result in behavioral sequelae that affect both the quality of life and longevity of patients, with consequent heavy economic and mental burden globally (Benjamin et al., 2017; Benjamin et al., 2018). In clinical practice, thrombolytic treatment remains the major therapeutic option for patients with ischemic stroke, but the use of recombinant tissue plasminogen activator (rt-PA) is limited by the potential risk of hemorrhagic transformation and its narrow time window (Davis et al., 2006). Therefore, medications with broad therapeutic window and evident neuroprotective efficacy are urgently needed.

Monoamines may play specific roles in the recovery of sensory, motor, and autonomic function. Monoamine neurotransmitters, such as dopamine (DA), 3,4-dihydroxyphenylacetic acid (DOPAC), and homovanillic acid (HVA), play an important role in the brain and changes in the concentration are observed in ischemic stroke. DA production is regulated by the activity of tyrosine hydroxylase (TH) and aromatic amino acid decarboxylase; DA is metabolized into DOPAC by monoamine oxidase (MAO). Furthermore, DOPAC is metabolized into HVA by catechol-*O*-methyl transferase (Soriano et al., 1997). In this process, TH is the key enzyme in DA biosynthesis (Calvo et al., 2011). Following ischemic stroke, changes have been observed in vulnerable components of the brain, including the substantia nigra pars (SN), the globus pallidus (Gp), and caudate putamen (CPu) of the striatum (Lin et al., 2010; Shrivastava et al., 2012; Adam et al., 2013). The SN is located below the thalamus and is divided into two functionally different components: the pars reticulata (SNpr) and the pars compacta (SNpc). The SNpr is mainly composed of GABAergic-type fusiform neurons and is innervated by the ipsilateral Gp. The SNpc is composed of rich dopaminergic neurons projecting to the ipsilateral CPu nucleus. The CPu is the structure responsible for a wide variety of behavioral functions and is highly vulnerable to ischemia (Globus et al., 1988; Alexander et al., 1990; Sabogal et al., 2014). Lesions in the striatum and transient global ischemia cause significant loss of dopaminergic substantia nigra neurons (Ichitani et al., 1991; Burke et al., 1992; Gower and Tiberi, 2018). In addition, striatal infarction downregulates TH immunoreactivity following ischemia (Yamada et al., 1996; Li et al., 2016; Mao et al., 2017).

*Rhodiola rosea* L. is a common genus of the family Crassulaceae, which is native to Asia, Europe, and North America (Panossian et al., 2010; Anghelescu et al., 2018). *R. rosea* L. has a long history of use as an "adaptogen" for fighting stress and improving physical and mental performance in healthy people (Panossian et al., 2010). Generally, *R. rosea* extracts have been used to treat a wide variety of common conditions and complex diseases such as Alzheimer’s disease, pains, cardiovascular disease, cognitive dysfunctions, high-altitude sickness, cancer, and mood disorders (Panossian et al., 2010; Lekomtseva et al., 2017; Li et al., 2017; Concerto et al., 2018; Jowko et al., 2018). The main bioactive compound in *R. rosea* L. is salidroside (molecular formula: C_14_H_20_O_7_; molecular weight: 300.31; CAS registry number: 10338-51-9; PubChem CID: 159278) (Zhong et al., 2018). Results from recent studies have revealed that salidroside has various neuropharmacological effects, including action against Alzheimer’s disease, Parkinson’s disease, stroke, depression, traumatic brain injury, and Huntington’s disease; it can also improve cognitive function, treat addiction, and prevent epilepsy (Zhong et al., 2018). According to previous studies, its underlying mechanisms of anti-stroke action are due to inhibition of apoptosis, regulation of oxidative stress, and suppression of inflammation with good efficiency and low toxicity (Zhong et al., 2018). Although some studies have reported the anti-stroke effects of salidroside and have suggested underlying mechanisms, more experimental data are needed to support these effects before salidroside is applied to the clinic.

Owing to the increasing potential benefit of salidroside in treating ischemia, there is a growing demand in understanding its therapeutic basis. In the present study, we assessed the extracellular effect of acute administration of salidroside on monoamines, including DA, DOPAC, and HVA in the striatum of anesthetized rats using regional brain microdialysis with high-performance liquid chromatography coupled with electrochemical detection (HPLC-ECD) and synthetically characterized the area under the curve (AUC). Subsequently, neurobehavioral tests were performed to document the effect of salidroside on cerebral ischemia/reperfusion. In addition, MAO and TH were detected in the serum. TH immunostaining was analyzed in the CPu and SNpc to evaluate the effect of salidroside on the dopaminergic system. This study aimed to further reveal the basis of action and the neurochemical modulatory mechanism of salidroside in the dopaminergic system in a rat model of ischemia/reperfusion. In this study, we extended an initial study and investigated the underlying anti-stroke mechanisms of salidroside, focusing on the dopaminergic system in the rat brain.

## Materials and Methods

### Chemicals and Reagents

The drug used in the present study was salidroside (molecular formula: C_14_H_20_O_7_; CAS registry number: 10338-51-9, Nanjing Goren Bio-technology Co., LTD, Nanjing, China) and the purity was above 99%. The standard dopamine hydrochloride was purchased from Sigma-Aldrich (Steinheim, Germany), while the standard DOPAC and HVA were purchased from Shanghai Aladdin Bio-Chem Technology Co., Ltd. (Shanghai, China). Sodium dihydrogen orthophosphate dehydrate HPLC electrochemical grade was from Fisher Scientific (Leicestershire, United Kingdom). Acetonitrile, trimethylamine, and *o*-phosphoric acid for HPLC were from Fisher Scientific (Geel, Belgium). Sodium 1-octanesulfonate for ion-pair chromatography and sodium chloride for HPLC were supplied by Shanghai Macklin Biochemical Co., Ltd. (Shanghai, China). Ethylenediaminetetraacetic acid (EDTA) disodium salt dehydrate was purchased from Shanghai Aladdin Bio-Chem Technology Co., Ltd. (Shanghai, China). The other reagents, sodium chloride, potassium chloride, calcium chloride, and magnesium chloride hexahydrate for analytical grade, were purchased from Sinopharm Chemical Reagent Co., Ltd. (Shanghai, China). Ultrapure water (18.2 MΩ Nanopure UV/UF, Thermo Scientific, Marietta, USA) was used throughout the study.

### Experimental Animals

Seven- to 8-week-old male Sprague-Dawley rats (280 ± 10 g) were purchased from Shanghai SLAC Laboratory Animal Co., Ltd. (Shanghai, China). The rats were housed at a temperature between 22 and 26°C with constant humidity of 45-70% and a 12-h light/dark cycle. The rats, which had *ad libitum* supply of food and tap water throughout the period of experiment, were housed in groups of four or five in standard polypropylene cages. The animals were acclimatized to the environment for more than a week before the start of experiment. All protocols were performed in accordance with the National Institutes of Health for Care and Use of Laboratory Animals and were approved by the Laboratory Animal Welfare and Committee of Ethics of the Fujian Academy of Traditional Chinese Medicine. All rat behavioral experiments were performed between 08:30 and 17:00.

Before the start of the experiments, based on the weight, 50 rats were randomly divided into five matched groups (10 in each group), namely: sham-operated, MCAO + saline solution, MCAO + 20 mg/kg salidroside, MCAO + 40 mg/kg salidroside, and MCAO + 80 mg/kg salidroside. With the exception of the sham-operated, drug or saline solution was given *via* intraperitoneal (i.p.) injection 1 min before cerebral ischemia.

### Implantation of Microdialysis Probes and Guide Cannula

**Figure 1:**
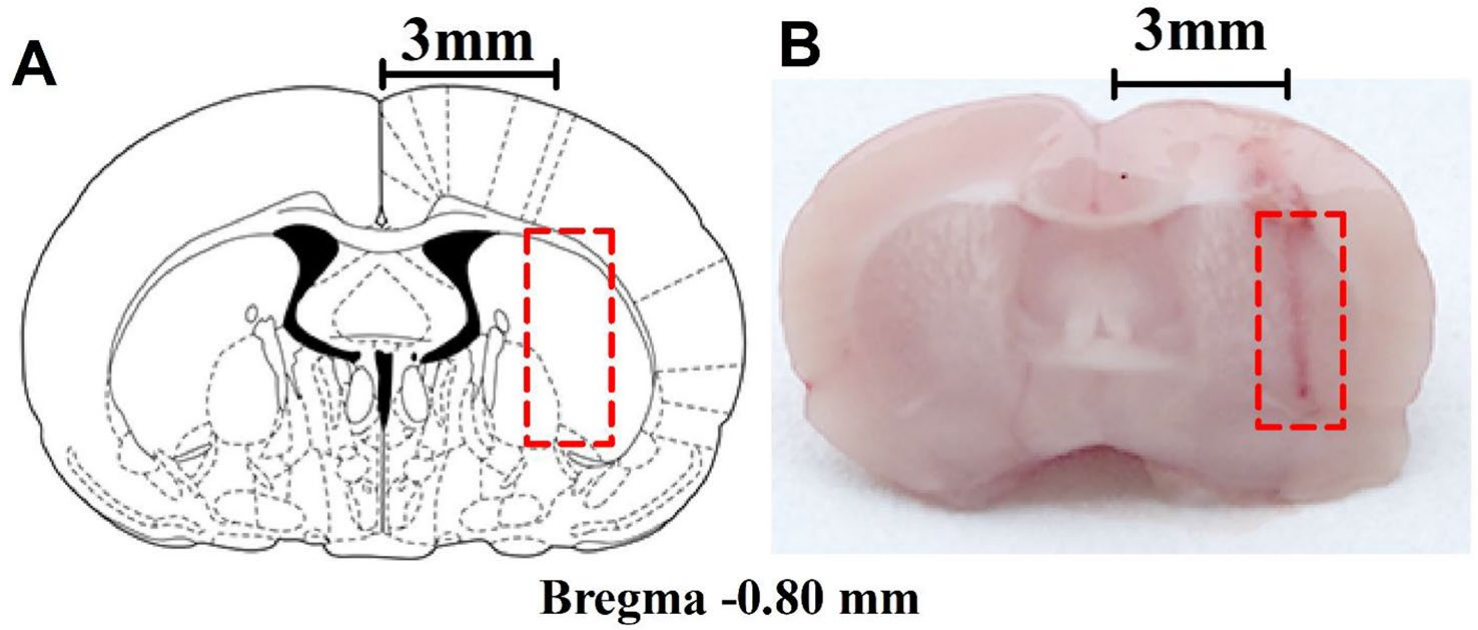
Anatomical placements of microdialysis probes. **(A)** The dialysis membrane was designed to be in the lateral part of the striatum (based on Paxinos and Watson, 2005). **(B)** Histological location of microdialysis examined in the brain. The *numbers* refer to the distance from bregma.

The microdialysis implantation has previously been described by Grabb et al. (1998). The rats were anesthetized with high-concentration isoflurane in the cage, and anesthesia was maintained with low-density continuous isoflurane using a nose cone; their rectal temperature was controlled at 37.5°C during surgery with a heating pad and monitored with a rectal thermistor. The head of an anesthetized rat was mounted in a stereotaxic apparatus (RWD Life Science Co., Ltd., Shenzhen, China) with a nose bar, and the surgical site was shaved and disinfected with iodophor. A midline incision was made through the skin to expose the surface of the flat-skull and a guide cannula (CMA 12 Guide Cannula, CMA Microdialysis, Solna, Sweden) was implanted into the right striatum through a bur hole in a dorsal position over the striatum at the following coordinates relative to bregma: anterior: 0.8 mm; lateral (L): 3 mm; dorso-ventral: 3.5 mm, according to the rat brain atlas by Paxinos and Watson (Paxinos and Watson 2005). The guide cannula was secured with anchor screws (CMA anchor screws, CMA Microdialysis, Solna, Sweden) and dental cement. After surgery, the rats were left to recover for 4-6 days in separate cages. The position of the guide cannula was observed histologically (**Figures 1A, B**) after the behavioral experiments, and rats with improper placement were not included in the statistical analysis.

### Microdialysis Procedure

**Figure 2:**
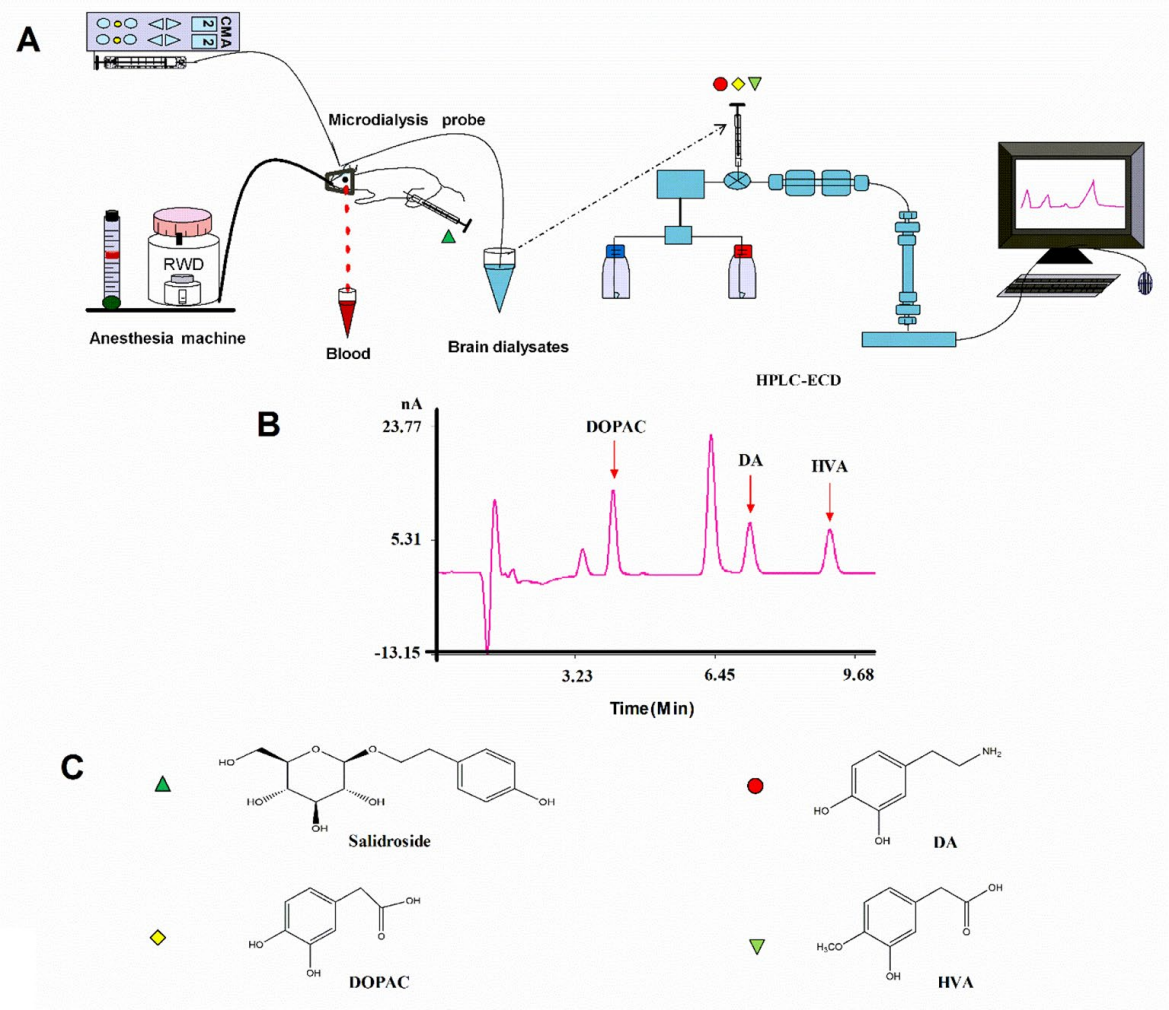
**(A)** Schematic illustration of the experimental protocols. **(B)** HPLC-ECD analysis of DA, DOPAC, and HVA in striatal fluid samples. **(C)** Chemical structures of salidroside, DA, DOPAC, and HVA. *HPLC-ECD* high-performance liquid chromatography coupled with electrochemical detection, *DA* dopamine, *DOPAC*, 3,4-dihydroxyphenylacetic acid, *HVA* homovanillic acid.

After recovery, the rats were fasted for 12 h and then anesthetized with isoflurane and the previous steps were followed throughout the experiment (**Figure 2**). A microdialysis probe (CMA 12 Elite, CMA Microdialysis, Solna, Sweden) was inserted through the guide cannula and was connected to the sampling system comprising a syringe micro-infusion pump (CMA 402, CMA Microdialysis, Solna, Sweden) and a sample auto-collector (MAB 85, Minnesota, USA) set at 4°C. The probes were perfused with a modified Ringer’s solution (140 mM NaCl, 4.0 mM KCl, 1.2 mM CaCl_2_, and 1.0 mM MgCl_2_, pH 7.3) at the rate of 2.0 µl/min. Following a 2-h stabilization period, a baseline sample was collected at 20 min. Then, middle cerebral artery occlusion (MCAO) was induced, and the surgery lasted for about 20 min. All microdialysis perfusates were collected into 4 µl of 1 M perchloric acid in microcentrifuge tubes every 20 min; dialysates were frozen and stored at −80°C until they were analyzed. The rectal temperature of the rats was controlled at 37.5°C throughout the microdialysis procedure. After the experiment, rats recovered from anesthesia before the behavioral tests.

### Middle Cerebral Artery Occlusion

MCAO was carried out as previously described (Han et al., 2015). In brief, a middle incision was performed in order to expose the right common carotid artery (CCA); branches of the external carotid artery (ECA) and internal carotid artery (ICA) were electro-coagulated. Then, the ECA was ligated with 5-0 braided silk sutures, while the CCA was temporarily clamped with a microsurgical clip. Occlusion of the CCA was induced *via* a small puncture by advancing a silicone-coated monofilament (Guangzhou Jialing Biotechnology Co., Ltd., Guangzhou, China; 3600) along the ICA for 17-18 mm from the bifurcation of the ECA. Rats allocated to the sham-operated group underwent an identical surgical procedure, but the CCA was not occluded. The filament was left there for 120 min until reperfusion. In general, mortality was not observed during the MCAO, but mortality after surgery was 6.6% (3/50), and these rats were excluded from the study.

### Biochemical Analysis of Striatal Perfusate

DA, DOPAC, and HVA in the striatal perfusate were determined using HPLC-ECD. The HPLC-ECD device used was the ALEXYS neurotransmitter analyzer from Antec (Zoeterwoude, the Netherlands), which consists of an OMD valve option Valco manual injector, an LC 110S pump, an OR 110 degasser unit, and the DECADE II electrochemical detector. The injection volume is 20 µl. Separations were performed on a QUATTRO 3 HPLC C_18_ column (150 × 2.1 mm, 3 µm) from Chrom 4 (Thuringen, Germany). The detector was equipped with a SenCell (2-mm glassy carbon working electrode, Ag/AgCl reference electrode, spacing distance 50 µm). Instrument control and data acquisition were carried out using Clarity Chromatography Software version 6.2.0 of Data Apex (Prague, Czech Republic).

The chromatographic conditions were based on the HPLC-ECD method described by Sarre et al. (Sarre et al., 1997). The mobile phase consists of 92.5% (*v*/*v*) of a buffered aqueous solution (75 mM sodium dihydrogen orthophosphate dehydrate, 1.7 mM sodium 1-octanesulfonate, 1.416 mM acetonitrile, 7.142 M triethylamine, and 0.01 mM Na_2_EDTA; pH was adjusted to 3.8 with *o*-phosphoric acid) and 7.5% (*v*/*v*) acetonitrile. The injection volume was 20 µl, the mobile phase flow rate was 300 µl/min, the separation and detection temperatures were set at 35°C, and the detection potential was +700 mV versus Ag/AgCl.

### Neurobehavioral Tests

The modified neurological severity score (mNSS) was used to assess the animals’ motor, sensory, balance, and reflex behaviors. In this mNSS, 1 score point is awarded for the inability to perform the test or for lack of reflex (Chen et al., 2001a; Chen et al., 2001b). Thus, a higher mNSS score correlates with a more severe injury. The neurological function was graded on a scale of 0 to 14 (normal score: 0; maximal deficit score: 14) (Mora-Lee et al., 2011).

The balance beam test was performed at the same time as the mNSS to assess the ability of rats to maintain balance and motor function while walking along an elevated and narrow strip of wood (80 × 1.5 cm) (Fan et al., 2018). A six-point scale was adopted in the test as follows: 0 point, balances with steady figure (> 60 s);1 point, grasps side of the beam; 2 points, hugs beam and one limb falls down from beam; 3 points, hugs beam and two limbs fall down beam, or spins on beam (> 60 s); 4 points, attempts to balance on the beam but falls off (> 40 s); 5 points, attempts to balance on the beam but falls off (> 20 s); 6 points, falls off with no attempt to balance or hang on to the beam (< 20 s) (Chen et al., 2001a).

The foot fault test assessed the sensorimotor coordination of the forelimbs (Horiquini Barbosa et al., 2016). Rats were placed on an elevated grid (45 × 30 cm, with 2.5 × 2.5-cm diameter openings, 8 cm higher than the solid base floor) and allowed to explore the grid for 3 min. The rats tended to place their paws on the wire frame when moving on the grid. When a rat places the forepaw incorrectly on the grid and the forelimb falls through or slips between the wires, it is counted as a fault. The percentage of foot faults of the left forepaw out of the total steps was calculated.

All these tests were evaluated 24 h after MCAO surgery by an investigator who was blinded to the experimental groups.

### Tyrosine Hydroxylase and Monoamine Oxidase ELISA Assay

For the measurement of TH and MAO levels, orbital blood was collected after the microdialysis procedure. The serum TH and MAO enzyme concentration were determined using a commercial ELISA kit (TSZ Biological Trade Co., Ltd., USA). According to the manufacturer’s instructions, the micro-ELISA strip-plate provided in this kit had been pre-coated with an antibody specific to TH or MAO. Standards and samples were added to the appropriate micro-ELISA strip-plate wells and combined to their specific antibodies. Then, samples were incubated with a streptavidin-horseradish peroxidase (HRP)-conjugated antibody specific for TH or MAO added to each well. Free components were washed away. The chromogen solution was added to each well, turning them blue. After the addition of the stop solution, the wells turned yellow. The optical density (OD) was measured at 450 nm. The OD value, which is proportional to the concentration of TH or MAO, was calculated by the OD of the samples to a standard curve.

### Tyrosine Hydroxylase Immunohistochemical and Immunofluorescence Staining

Following the neurobehavioral tests, rats were anesthetized with chloral hydrate (10%, *w*/*v*) and were intracardially perfused with 500 ml of 0.9% physiological saline, followed by 350 ml of 4% paraformaldehyde. Rats were decapitated and their brains were fixed in 4% paraformaldehyde overnight. After the brain tissues were dehydrated with 30% sucrose in paraformaldehyde, serial coronal sections (30 µm) of the CPu and SNpc (CPu bregma −1.00 to 1.40 mm; SNpc: bregma −5.2 to −5.6 mm; Paxinos and Watson 2005) were obtained using a cryostat (CM1900, Leica Microsytems, Germany) at −20°C. To prevent penetration injury in the ipsilateral striatum because of microdialysis probe implantation, the CPu region in the test was set slightly behind the microdialysis probes.

For immunofluorescence staining, the free-floating sections were incubated in 5% bovine serum albumin blocking reagent for 1 h to block nonspecific binding (1:200, Beyotime Biotech Inc., Shanghai, China). The brain sections were then incubated in rabbit anti-tyrosine hydroxylase (1:1,000, Abcam, ab75875, USA) overnight at 4°C. Binding was visualized using Alexa Fluor 488-conjugated goat anti-rabbit IgG (1:200, Beyotime Biotech Inc., Shanghai, China), and the nuclei were stained with 4¢,6-diamidino-2-phenylindole (DAPI, Beijing Solarbio Science & Technology Co., Ltd., Beijing, China). Fluorescence intensity was measured using ImageJ software (National Institutes of Health, Bethesda, MD, USA).

For immunohistochemical staining, the slices were incubated in 3% hydrogen peroxidase for 10 min to inhibit endogenous peroxidase activity. Then, sections were reacted with a rabbit TH polyclonal antibody (1:1,000, Abcam, ab75875, USA) at 4°C overnight. The next day, sections were incubated with HRP-polymer anti-rabbit IgG working solution (Maixin Biotech. Co., Ltd., Fuzhou, China) for 15 min and were stained with diaminobenzidine (DAB) for 5 min. Subsequently, sections were cleared with xylene and mounted with Canada balsam. Quantification of immunoreactivity in the areas tested was determined using a 10 × objective for CPu and SNpc. Photomicrographs of the immunohistochemical assays were also analyzed using ImageJ software (National Institutes of Health, Bethesda, MD, USA).

### Statistical Analysis

All data were analyzed with SPSS 16.0 (SPSS Inc., Chicago, IL, USA). Numerical values are reported as the mean ± standard error of the mean (SEM). A value of *p* < 0.05 was considered statistically significant.

Concentrations of DA, DOPAC, and HVA were expressed as a percentage of the last two baseline dialysate levels and analyzed using repeated measures two-way ANOVA followed by Dunnett T3 multiple comparisons with time as the repeated measure factor and drug treatment as the between-group factor. As the Mauchly’s test indicated that sphericity was violated, the degrees of freedom were corrected using Greenhouse-Geisser estimates of sphericity. Other data analyses of different groups were performed using nonparametric tests (Kruskal-Wallis followed by Mann-Whitney *U* tests), one-way analysis of variance [ANOVA: Dunnett T3 multiple comparison tests and least significant difference (LSD) test].

## Results

### Release of DA, DOPAC, and HVA in Striatal Microdialysis Fluid on Salidroside-Treated Cerebral Ischemia/Reperfusion Rats

**Figure 3:**
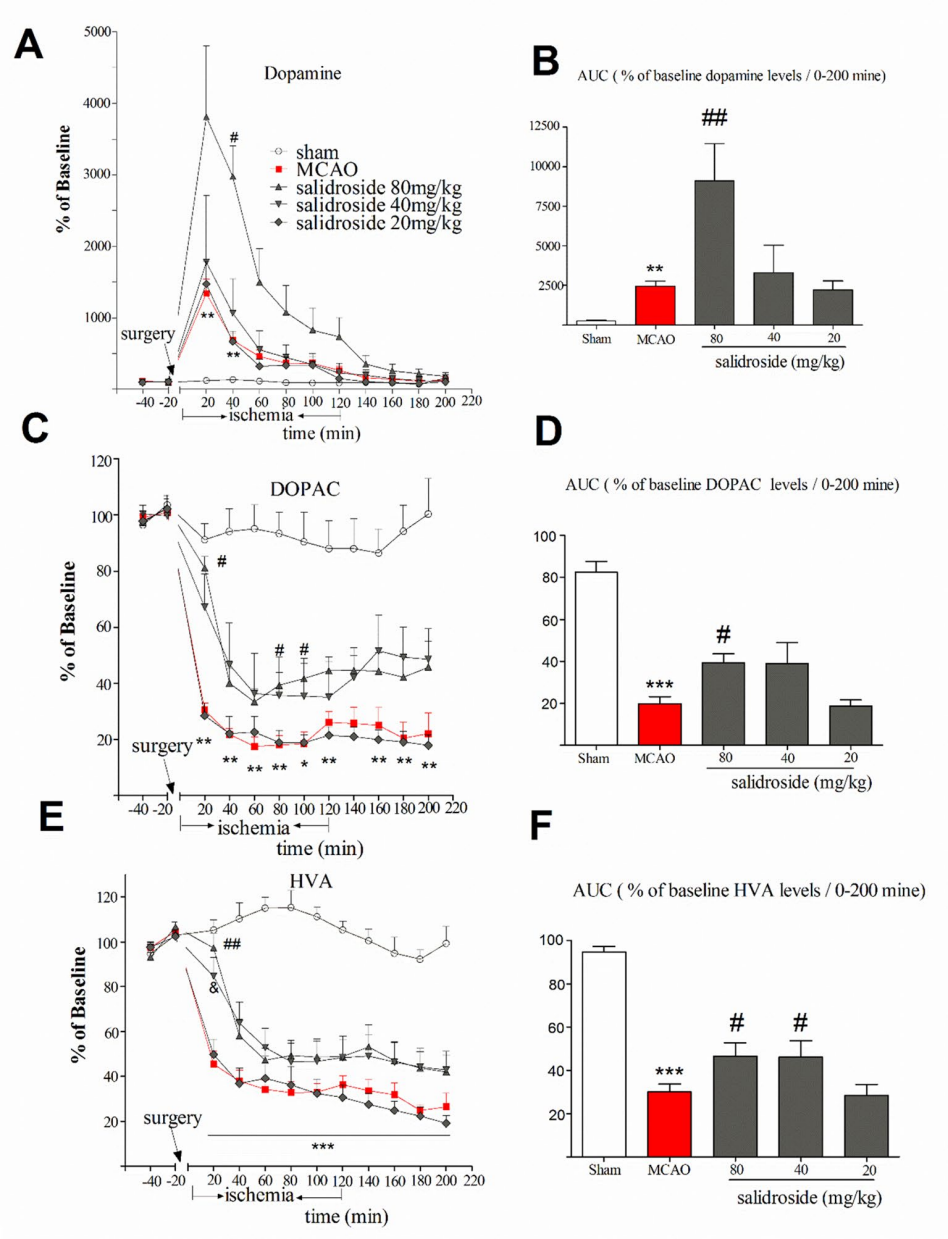
Time-course percentage changes in extracellular DA, DOPAC, and HVA levels in the striatum during cerebral ischemia/reperfusion under a single i.p. injection of saline solution or salidroside (80 mg/kg, 40 mg/kg, and 20 mg/kg). Extracellular levers of DA, DOPAC, and HVA in the striatum were monitored for 4 h; their levels are expressed as percentage of baseline **(A**, **C**, **E)**. The area under curve (AUC) for extracellular percentage of baseline DA, DOPAC, and HVA levels was calculated from their release curves and was plotted in the striatum over a period of 200 min (0-200 min) **(B**, **D**, **F)**. The *arrow* indicates the surgery of MCAO. Each *column* represents the mean and the SEM, *n* = 7. **p < 0.01, ***p < 0.01 compared to the sham group; ^#^ *p* < 0.05, ^##^ *p* < 0.01, ^&^ *p* = 0.058 compared to the MCAO group. *AUC* area under the curve, *DA* dopamine, *DOPAC* 3,4-dihydroxyphenylacetic acid, *HVA* homovanillic acid, *MCAO* middle cerebral artery occlusion, *SEM* standard error of the mean.

The anti-stroke effects of salidroside on striatum monoamines levels are depicted in **Figure 3**. In this study, the mean of DA, DOPAC, and HVA concentrations of two samples prior to the MCAO surgery were considered as baseline levels for each probe, respectively. The time-course graphs present the percentage of changes in extracellular DA, DOPAC, and HVA from the baseline levels (**Figures 3A, C, E**). The AUC calculated from the DA, DOPAC, and HVA release curves was plotted using their extracellular percent levels in the striatum over a period of 200 min (0-200 min) (**Figures 3B, D, F**).

Basal level of DA was 35.6 ± 5.46 µM. As shown in **Figure 3A**, significant effects of treatment (*F*_4, 30_ = 5.871, *p* < 0.001) and time (*F*_11, 330_ = 31.257, *p* < 0.001), as well as a significant interaction between treatment and time (*F*_44, 330_ = 5.338, *p* < 0.001), were observed. Compared with sham group, MCAO rats had significantly increased levels of DA at 20 min (*p* < 0.01) and 40 min (*p* < 0.01) time after the surgery and remained at a high level for about 120 min. The maximum increased rate of the DA levels of salidroside-treated [20, 40, and 80 mg/kg body weight (bw), i.p.] were 3810.8 ± 630.70%. At 40 min time, salidroside (80 mg/kg) significantly increased the levels of DA compared to the MCAO rats (*p* < 0.05). Moreover, salidroside-treated rats (80 mg/kg) remained at a high level for about 140 min. The AUC data for extracellular percent DA levels in the striatum revealed a significant treatment effect between subjects (*H*_4_ = 24.299, *p* < 0.001). As shown in **Figure 3B**, the MCAO-operated rats had significantly increased DA levels after treatment compared with the sham-operated rats (*p* < 0.01). The administration of salidroside (80 mg/kg) further increased the DA levels (*p* < 0.01 vs. the vehicle group). These findings suggested that intraperitoneal injection of salidroside can further improve the level of DA.

The basal level of DOPAC was 2,048.0 ± 114.50 µM. We examined the effect of salidroside in the striatum on the extracellular DOPAC levels (**Figures 3C, D**). A two-way ANOVA performed on the percent changes in DOPAC levels revealed a significant treatment effect between subjects (*F*_4, 30_ = 21.797, *p* < 0.001) and that DOPAC significantly changed with time (*F*_11, 330_ = 76.224, *p* < 0.001). Furthermore, there was a significant interaction between treatment and time (*F*_44, 330_ = 5.050, *p* < 0.001). *Post hoc* Dunnett’s T3 tests revealed that MCAO treatment significantly decreased DOPAC levels (*p* < 0.01 vs. the sham group) from postoperative 20 min time (30.4 ± 6.6%) to the end of the entire observation period, reaching a maximum rate of decrease in DOPAC levels of 17.5 ± 8.3%. In these experiments, administration of salidroside (80 mg/kg bw, i.p.) significantly reversed DOPAC levels (*p* < 0.05) compared to the effect of the vehicle at 20, 80, and 100 min time. The percent changes at 20 min time was 81.0 ± 6.6% of basic after 80 mg/kg salidroside administration (*p* < 0.05 vs. the MCAO group). A significant treatment effect on the AUC data for extracellular percent DOPAC levels was observed between the rats (*F*_4, 30_ = 20.595, *p* < 0.001).The AUC data was significantly decreased in the MCAO rats compared to the sham rats (*p* < 0.001). Salidroside-treated rats showed a significant increase at 80 mg/kg (*p* < 0.05) relative to the MCAO-treated rats. Together, these findings suggest that salidroside may reverse the decrease in the DOPAC level.

The basal level of extracellular HVA was 826.40 ± 58.97 µM. A two-way ANOVA on the ipsilateral striatum HVA levels demonstrated a significant treatment effect (**Figure 3E**) between subjects (*F*_4, 30_ = 5.871, *p* < 0.001) and time (*F*_11, 330_ = 104.685, *p* < 0.001) and a significant interaction between treatment and time (*F*_44, 330_ = 10.138, *p* < 0.001). *Post hoc* Dunnett’s T3 tests showed MCAO treatment significantly decreased the level of HVA compared to sham group (*p* < 0.001), demonstrating the significant development of low HVA levels from the 20-min time (45.5 ± 6.5%) after MCAO surgery to the end of the observation period (*p* < 0.001). Administration of salidroside (80 mg/kg bw, i.p.) significantly elevated HVA level at 20 min time (*p* < 0.01 vs. vehicle control) and reached the maximum protective effect (97.3 ± 6.5%). There were also significant differences between the groups in the AUC data of the HVA (F_4, 30_ = 26.085, *p* < 0.001). As shown in **Figure 3F**, MCAO-operated rats had significantly reduced HVA levels (*p* < 0.001 vs. vehicle control). Salidroside (80 and 40 mg/kg bw, i.p.) treated rats showed a mild increase in the AUC data of HVA levels in the striatum relative to MCAO vehicle rats (*p* < 0.05). These data suggest that salidroside alleviates the effect of cerebral ischemia/reperfusion by up-regulating HVA level in the striatum.

### Salidroside Improves Neurobehavioral Impairment Following Focal Cerebral Ischemia/Reperfusion

**Figure 4:**
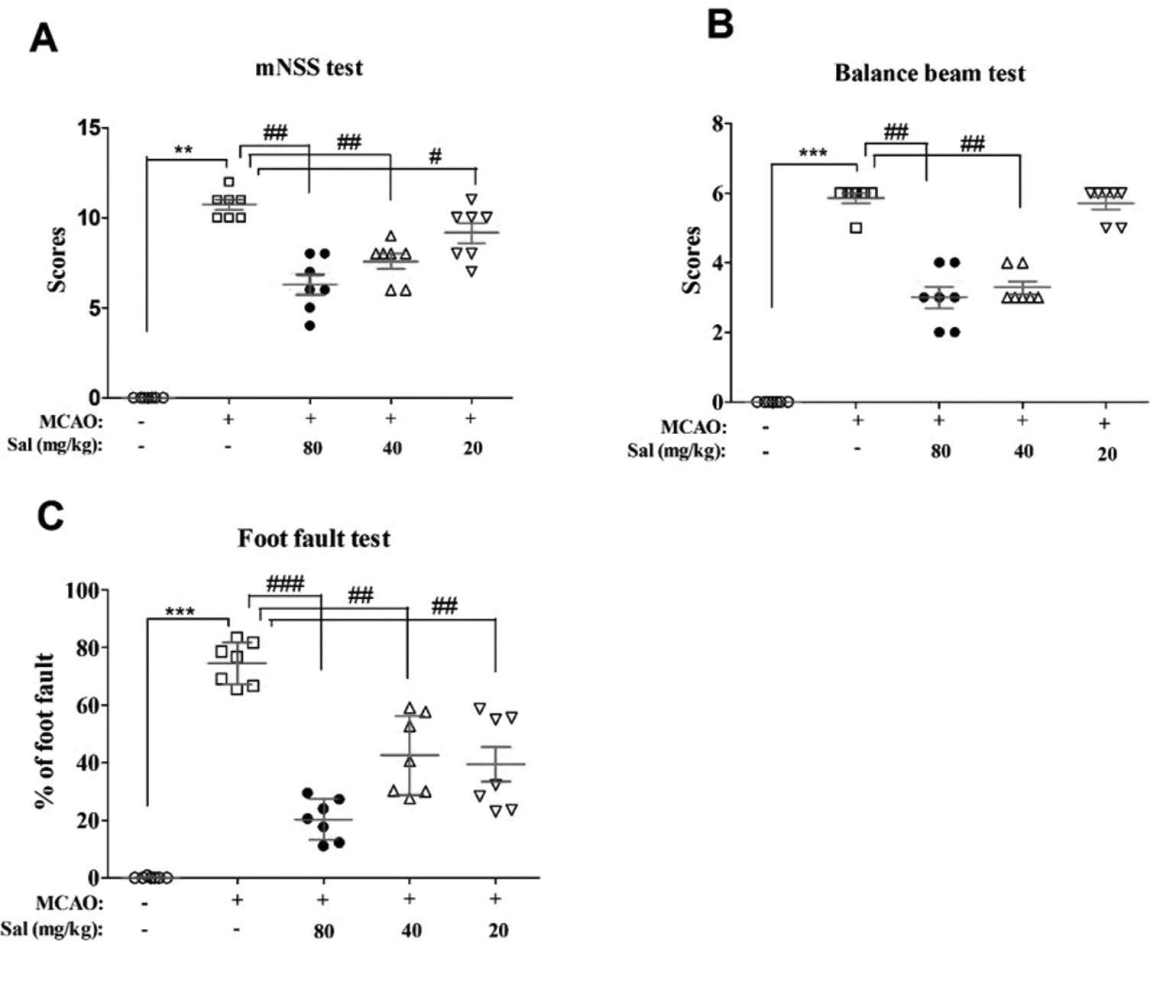
Salidroside improves neurobehavioral impairment after MCAO. Rats that were treated with 80, 40, and 20 mg/kg salidroside or saline water prior to MCAO exhibited significant change in the mNSS test **(A)**, balance beam test **(B)**, and foot fault test **(C)**. Data are expressed as the mean ± SEM, *n* = 7. **p < 0.01, ***p < 0.001 compared to the sham group; ^#^ *p* < 0.05, ^##^ *p* < 0.01, ^###^ *p* < 0.001 compared to the MCAO group. *MCAO* middle cerebral artery occlusion, *mNSS* modified neurological severity score, *SEM* standard error of the mean.

Effects of salidroside on neurobehavioral impairments were examined using mNSS, balance beam test, and foot fault test (**Figure 4**). There were significant differences between the experimental groups in mNSS scores (*H*_4_ = 28.522, *p* < 0.001), balance beam test scores (*H*_4_ = 31.137, *p* < 0.001), and the foot fault test (*F*_4, 30_ = 48.385, *p* < 0.001). In these experiments, MCAO rats had significantly increased neurological deficit and balance beam test scores and percent in the foot fault test (mNSS scores: *p* < 0.01, **Figure 4A**; balance beam test scores: *p* < 0.001, **Figure 4B**; foot fault test: *p* < 0.001, **Figure 4C**) compared to those of the sham group. Salidroside significantly decreased these measures compared with MCAO (mNSS scores: *p* < 0.01 for salidroside of 40 and 80 mg/kg, *p* < 0.05 for salidroside of 20 mg/kg, **Figure 4A**; balance beam test scores: *p* < 0.01 for 40 and 80 mg/kg, **Figure 4B**; foot fault test: *p* < 0.001 for 80 mg/kg, *p* < 0.01 for 40 and 20 mg/kg, **Figure 4C**). The anti-stroke effects of salidroside were dose-dependent (*H*_2_ = 8.643, *p* < 0.05 for mNss; *H*_2_ = 14.653, *p* < 0.01 for balance beam test; *F*_2, 18_ = 6.083, *p* < 0.05 for foot fault test). These data suggest that salidroside may possess the ability to ameliorate neurobehavioral impairment after cerebral ischemia/reperfusion.

### Salidroside Increases Monoamine Oxidase in Serum Following Focal Cerebral Ischemia/Reperfusion

**Figure 5:**
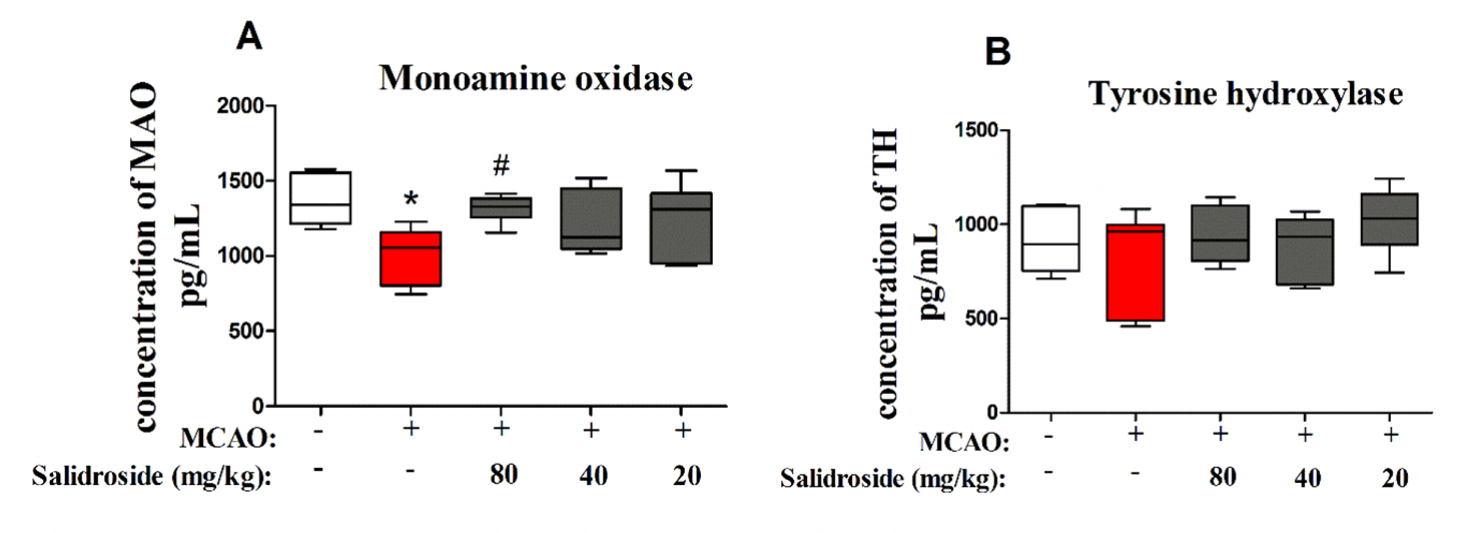
Salidroside treatment modulates MAO in the serum of MCAO-induced rats. Salidroside (20, 40, and 80 mg/kg) or vehicle was i.p. administered before the surgery. After the microdialysis procedure, the levels of MAO **(A)** and TH **(B)** were determined using ELISA. Data are presented as the mean ± SEM (*n* = 7 rats per group). *p < 0.05 compared with the sham group; ^#^ *p* < 0.05 compared to the MCAO group. *MAO* monoamine oxidase, *TH* tyrosine hydroxylase, *MCAO* middle cerebral artery occlusion, *ELISA* enzyme-linked immunosorbent assay, *SEM* standard error of the mean.

To further examine the dopaminergic system after salidroside treatment in MCAO-induced stroke, MAO and TH were determined using ELISA. There were significant differences between groups in MAO levels (*F*_4, 30_ = 3.804, *p* < 0.05; **Figure 5A**). After 36 h of cerebral ischemia/reperfusion injury, the level of MAO was significantly decreased (*p* < 0.05) and markedly increased after treatment with 80 mg/kg salidroside (*p* < 0.05). However, the TH level was not significantly altered in the groups (*F*_4, 30_ = 1.253, *p* > 0.05; **Figure 5B**). These data suggest that salidroside alleviates the effects of stroke by upregulating the MAO level in serum.

### Salidroside Increases Tyrosine Hydroxylase Immunoreactivity in the CPu Following Focal Cerebral Ischemia/Reperfusion

**Figure 6:**
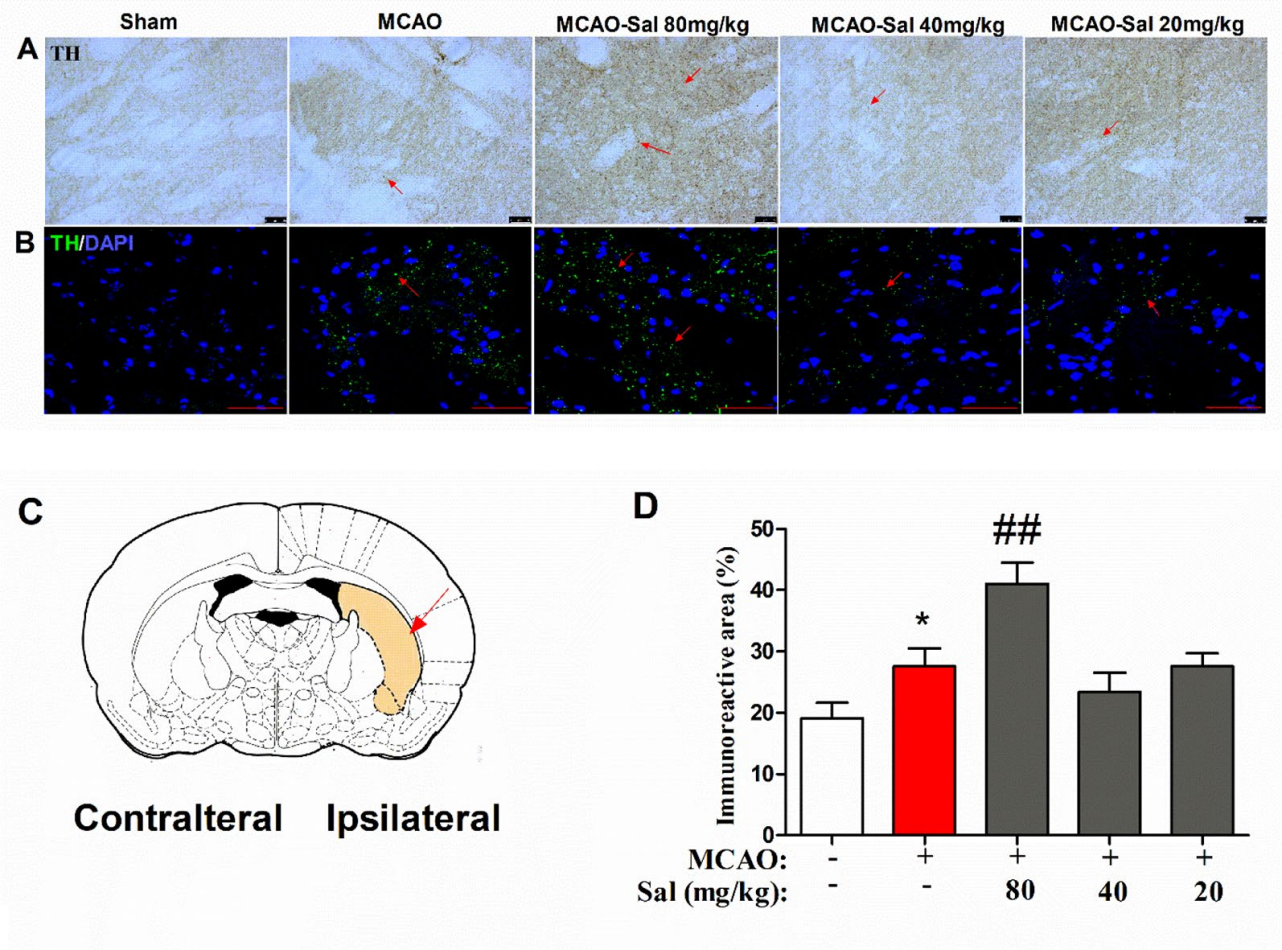
Effects of salidroside on immunoreactivity of TH in the CPu after MCAO in rats. **(A)** TH immunohistochemical staining in the ipsilateral CPu. *Arrows* indicate TH-positive expression. *Scale bar*, 50 µm. **(B)** TH immunofluorescence staining in the ipsilateral CPu. *Arrows* indicate TH-positive expression. *Scale bar*, 50 µm. **(C)** Schematic diagram of the rat brain. *Arrow* indicates CPu. **(D)** Immunoreactivity area percent of TH-positive expression in the CPu after cerebral ischemia/reperfusion in rats. Values are all represented as the mean ± SEM (*n* = 5 rats per group). *p < 0.05 compared with the sham group; ^##^ *p* < 0.01 compared to the MCAO group. *CPu* striatal caudate putamen, *MCAO* middle cerebral artery occlusion, tyrosine hydroxylase, *SEM*, standard error of the mean.

To further investigate the mechanism of monoamine neurotransmitters, the key release location of monoamine neurotransmitters in the CPu was assessed by immunohistochemical and immunofluorescence staining in salidroside-treated MCAO rats. As shown in **Figures 6A–D**, there were significant differences between the experimental groups (one-way ANOVA: *F*_4, 95_ = 8.411, *p* < 0.001). MCAO treatment significantly increased TH-positive expression after a 36-h cerebral ischemia/reperfusion injury (*p* < 0.05). Administration of salidroside (80 mg/kg bw, i.p.) further increased the TH-positive expression (*p* < 0.001). These data suggest that the salidroside-treated effect on monoamine neurotransmitters may be linked to an increase in TH in the CPu.

### Salidroside Increases Tyrosine Hydroxylase Immunoreactivity in the SNpc Following Focal Cerebral Ischemia/Reperfusion

**Figure 7:**
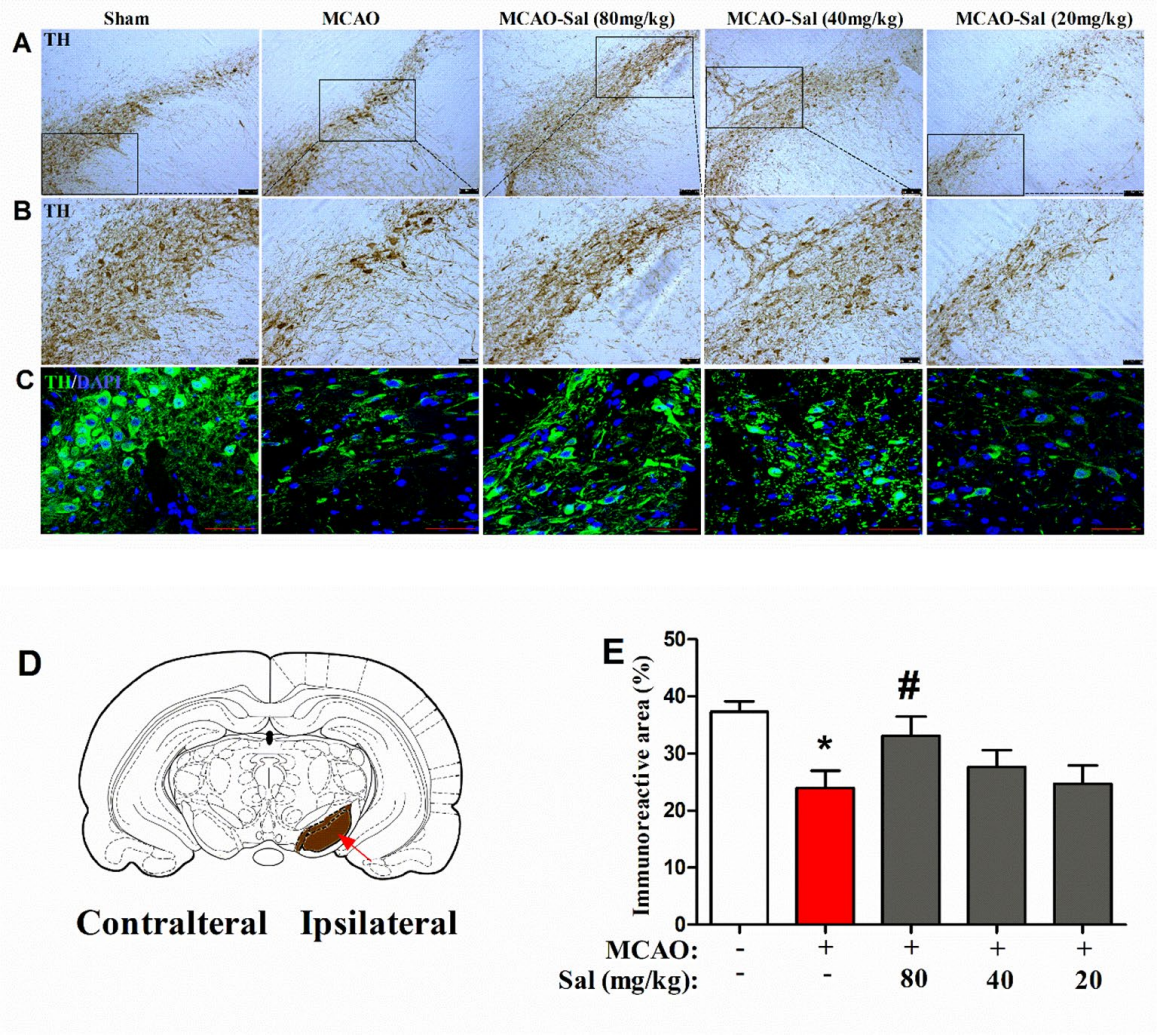
Effects of salidroside on TH immunoreactivity in the SNpc after MCAO in rats. **(A**, **B)** TH immunohistochemical staining in the dopaminergic mesencephalic nuclei of the ipsilateral SNpc. *Scale bar*, 100 µm **(A)** and 50 µm **(B)**. **(C)** TH immunofluorescence staining in the dopaminergic mesencephalic nuclei of the ipsilateral SNpc. *Scale bar*, 50 µm. **(D)** Schematic diagram of rat brain *Arrow* indicates SNpc. **(E)** Immunoreactivity area percent of TH-positive expression in the SNpc after cerebral ischemia/reperfusion in rats. Values are all represented as the mean ± SEM (*n* = 5 rats per group). *p < 0.05 compared with the sham group; ^#^ *p* < 0.05 compared with the MCAO group. *MCAO* middle cerebral artery occlusion, *SEM* standard error of the mean, *SNpc* substantia nigra pars compacta, *TH* tyrosine hydroxylase.

We observed the effects of salidroside administration on the expression of TH in the SNpc by immunohistochemical and immunofluorescence staining (**Figures 7A–E**). In the present study, a significant treatment effect on the TH immunoreactive area percent was observed between rats (one-way ANOVA: *F*_4, 95_ = 3.842, *p* < 0.01). As shown in **Figure 7E**, the TH immunoreactive area was significantly decreased in MCAO rats (*p* < 0.05 vs. the sham group). After two consecutive days of salidroside administration (80 mg/kg), the TH immunoreactive area percent in the SNpc of MCAO rats was further enhanced (*p* < 0.05 vs. the MCAO group). This finding implies that the increased TH immunostaining in the CPu of salidroside-treated MCAO rats may be related to an enhanced TH bioactivity in the rat SNpc.

## Discussion

In the present study, salidroside attenuated neurobehavioral impairment, which was induced by maintaining striatal monoamine neurotransmitter levels, their synthetic enzyme levels, and TH expression in the CPu and the SNpc after acute ischemic stroke. These findings are consistent with our hypothesis and suggest that the anti-stroke effect of salidroside is related to the dopaminergic system. Importantly, salidroside may provide an alternative treatment avenue of patients with abnormal dopaminergic system in acute ischemic stroke.

A growing number of studies have shown that salidroside has neuroprotective effects on focal cerebral ischemia/reperfusion-induced stroke. For example, studies have reported that salidroside increases the neurological severity scores and reduces the cerebral edema and infarction volume at doses of 12 or 24 mg/kg (Zou YQ et al., 2009; Shi et al., 2012; Han, 2013). Our previous studies demonstrated that salidroside displayed strong and specific neuroprotective properties in MCAO-induced stroke with treatment (50 mg/kg, administered 1 h after the operation) once daily for 1, 2, and 6 days, or with only a two-time administration at a dose of 30 mg/kg (administered immediately before cerebral ischemia and after reperfusion) (Han et al., 2015; Lai et al., 2015; Lai et al., 2018). In a previous study, we found salidroside (30 mg/kg) reduced infarct size in 2,3,5-triphenyltetrazolium chloride-stained brain slices and improved histological changes in the cortex and striatum as revealed by Nissl staining (Han et al., 2015). To further validate the effect of salidroside on cerebral ischemia/reperfusion, we used the balance beam test and foot fault test. The foot fault test is a very sensitive method for evaluating the sensorimotor coordination of performance after cerebral ischemia/reperfusion in rats, while the balance beam test is undoubtedly a more predictive assessment of hindlimb placing deficits (Schaar et al., 2010). Here, we demonstrated that salidroside ameliorated dose-dependently the neurobehavioral impairment at 20-80 mg/kg once daily for 2 days revealed using the above tests, confirming the reliability and validity of salidroside on neurobehavioral recovery. Although multifarious behavioral tests have been used to evaluate the effects of salidroside on neurological function in ischemic rats for the assessment of potential therapeutic treatment of stroke, understanding how salidroside affects additional neurological deficits, such as dexterity and sensory capacities, may be useful for developing future rehabilitation therapies for stroke patients.

In recent years, a growing number of studies have indicated the involvement of dopaminergic systems in stroke modulation (Gower and Tiberi, 2018). The results of microdialysis of the present study demonstrated that cerebral ischemia induced a robust and transient release of DA extracellularly in the striatum, which is consistent with previous studies (Globus et al., 1988; Tsukada et al., 2004; Li et al., 2010; Yoshimoto et al., 2014). Moreover, the dynamic variations of DOPAC and HVA were consistent with that reported previously (Globus et al., 1988). In the present study, we found that salidroside administration induced an additional increase in dopamine and reversed the reduction of both DOPAC and HVA. These results were similar to those reported recently, in which salidroside administration increased the levels of DA, DOPAC, and HVA in the striatum of brains with neurodegenerative disease (Zhang et al., 2016; Li and Chen, 2019). Under normal conditions, the dopaminergic system maintains a steady state; however, this fine balance is affected when any factor is disrupted and consequently induces serious physiological reactions (Defaix et al., 2018; Zeiler and Menon, 2018; Dyhrfort et al., 2019). The sharp increase in extracellular DA could probably have resulted from its ingression through an injured or damaged blood-brain barrier. Nevertheless, salidroside pretreatment has been reported to efficiently decrease the extent of damage in the blood-brain barrier (Han, 2013). Thus, it is likely that the rapid increase in extracellular DA may have resulted from its leakage from the intracellular compartment because of the marked stimulation of DA synthesis and/or reuptake inhibition. Normally, extracellular DA level is controlled by an energy-intensive efflux-influx transport cycle, which is balanced dynamically. ATP and ATP metabolites are required to keep DA compartmentalized inside synaptic terminals and to maintain the normal reuptake process. During ischemia, organ energy stores are depleted, which results in the inhibition of these energy-dependent transport and metabolism processes. Consequently, it leads to a reduced DA compartmentalization inside the synaptic terminals and a decreased reuptake process, causing an increase in the extracellular DA level (Fabre et al., 1999; Yu and Gao, 2017). Interestingly, in previous studies, salidroside was reported to promote energy metabolism after exercise-induced fatigue in mice (Jin et al., 2018). Hence, salidroside probably promotes energy metabolism to keep DA compartmentalized inside the synaptic terminals, which results in further compensatory elevation of the extracellular DA level. In the synaptic clefts, MAO is responsible for DA metabolism (Seif-El-Nasr et al., 2008). In addition, MAO is time-sensitive to cerebral ischemia. In rodents, MAO was reduced in the brain following 15-min ischemia, and the MAO activity never reached that of control levels during 7 days of reperfusion (Cvejic et al., 1980; Stanimirovic et al., 1994); In contrast, a different study showed that MAO was increased in the brain after a 22-h ischemia (Ansari et al., 2004). In clinical practice, during the acute period of ischemia stroke (3-5 days), MAO was increased in 25 patients compared to control levels, although the standard deviation was very high (Uzbekov et al., 2011). In our study, after 2-h ischemia, the MAO was decreased at 36 h, and salidroside (80 mg/kg) reversed the MAO levels. A significant association between MAO activity and neurological deficits has been revealed by dynamic monitoring of patients with ischemia stroke, and the duration of ischemia is a key factor for stroke rehabilitation (Uzbekov et al., 2011; Crunkhorn, 2018). The effect of the dynamic variation of salidroside on MAO during different durations of ischemia is unclear and requires further studies.

The striatal TH-positive neurons synthesize DA and release it into the synaptic cleft. In our study, we observed enlarged TH-positive expression with granular appearance after ischemia/reperfusion in the striatal CPu. These outcomes are in line with previous reports of enlarged granular TH terminals and large synaptophysin-positive dots after cerebral ischemia/reperfusion in the rat CPu (Sabogal et al., 2014). This phenomenon was considered as a compensatory function of the dopaminergic nerve terminals in response to the striatal neuron degeneration (Nemeth et al., 1991; Korematsu et al., 1993). An earlier study reported that salidroside upregulated the expression of dopamine transporter in the striatum in an animal model of a neurodegenerative disease (Li and Chen, 2019). Our data show more densely packed TH nerve dots after salidroside treatment, indicative of an elevated TH activity, in combination with a high DA concentration in the early periods after ischemia. It is also possible that salidroside treatment enables the repair of the axoplasma flow inside the axon terminals of the presynaptic neuron, promoting an anterograde accumulation of TH in the striatum. Therefore, it is suggested that further increase in TH may represent an adaptive change in response to extracellular release of DA.

**Figure 8:**
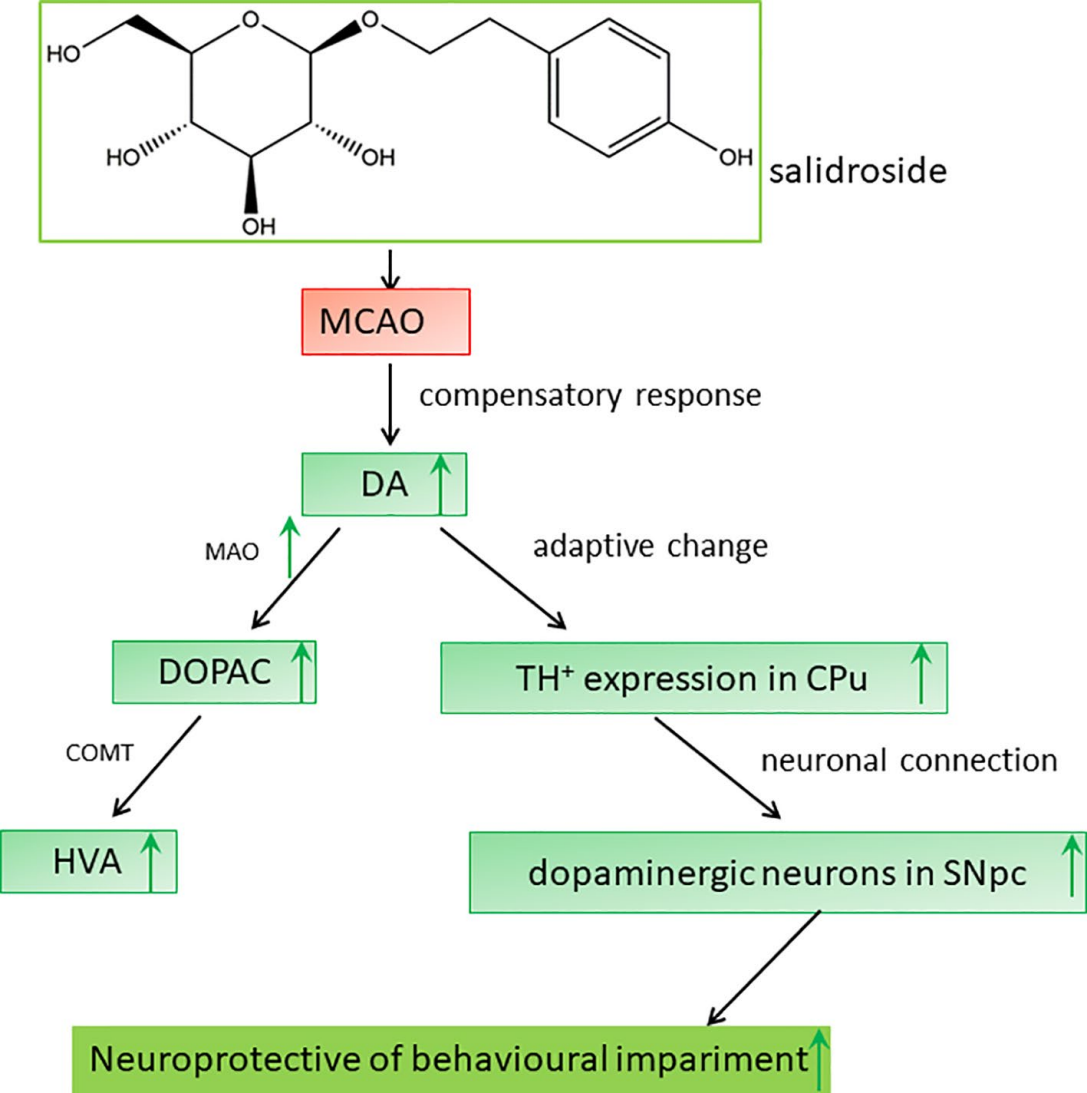
Proposed mechanisms of the effect of salidroside in MCAO rats. Salidroside attenuates MCAO-induced neurobehavioral impairment by increasing the levels of DA, DOPAC, HVA, and MAO and elevating TH-positive expression in the CPu and SNpc. *CPu* striatal caudate putamen, *DA* dopamine, *DOPAC* 3,4-dihydroxyphenylacetic acid, *HVA* homovanillic acid, *MAO* monoamine oxidase, *MCAO* middle cerebral artery occlusion, *SNpc* substantia nigra pars compacta, *TH* tyrosine hydroxylase.

Dopaminergic neurons are located in four regions of the rat brain: the ventral tegmental area (VTA), substantia nigra (SN), basal hypothalamus, and olfactory bulb. The SN neurons primarily project to the striatum along the nigrostriatal pathway (Bjorklund and Dunnett, 2007; Ott and Nieder, 2019). The dopaminergic pathway from the SNpc to the CPu is an important neuronal projection in the basal ganglia and plays a crucial role in the regulation of behavioral functions in stroke (Sun et al., 2012; Rodriguez-Grande et al., 2013; Rimmele et al., 2018). On immunohistochemical analysis, we observed a significant loss of dopaminergic neurons in the SNpc after ischemia/reperfusion, consistent to that reported previously (Ishibashi et al., 2004). Degenerative alterations in the ipsilateral SNpc have also been reported in clinical patients with neurodegenerative disorders (Lou et al., 2009; Nakajima et al., 2010; Robles, 2016). Several studies have demonstrated that salidroside potentially targets extracellular TH secretion in the SNpc (Zhang et al., 2016; Wu et al., 2018; Li and Chen, 2019). Indeed, our data showed a similar observation: salidroside treatment prevented dopaminergic neurons loss as displayed by the preservation of TH-positive cells in the rat SNpc. Ischemic injury to the SN, a central output nucleus of the basal ganglia, may lead to neurobehavioral impairment (Prinz et al., 2015; Zhang et al., 2015). Therefore, the neuroprotective effect of salidroside in the SNpc against ischemic impairment may constitute the foundation for the elevation of behavioral function. Consequently, we hypothesized that the anti-stroke effects of salidroside on MCAO-induced cerebral ischemia/reperfusion in rats may be involved in monoamine metabolism-induced modulation of the TH-positive cells in the CPu and the SNpc, which may be related to the function of the dopaminergic system in the brain (**Figure 8**). However, further studies are warranted to determine how salidroside influences the dopaminergic neuronal circuits between the CPu and the SNpc on cerebral ischemia/reperfusion models. In addition, more research is needed to introduce salidroside into clinical studies for treating patients with ischemic stroke.

## Conclusion

In conclusion, our results demonstrate that salidroside has a significant effect on cerebral ischemia/reperfusion-induced neurobehavioral impairment in rats; its neuroprotective effects are associated with the modulation of monoamine metabolism, which involves the dopaminergic system in the striatal CPu and the SNpc (**Figure 8**). Our study raises the possibility that salidroside will be efficacious in the management of neurobehavioral impairment related to the dopaminergic system in clinical patients with stroke.

## Data Availability Statement

All datasets analyzed for this study are included in the article/supplementary material.

## Ethics Statement

The animal study was reviewed and approved by Laboratory Animal Welfare and Committee of Ethics of the Fujian Academy of Traditional Chinese Medicine.

## Author Contributions

Z-FZ and JHa performed the experiments, analyzed the data, prepared the figures, and drafted the manuscript. J-ZZ, QX, J-YC, and KZ contributed to the experiments. JHu and L-DC conceived and designed the study. All authors read and approved the final manuscript.

## Funding

This work was supported by the National Natural Science Foundation of China (grant no. 81803870), and Fujian Provincial Science and Technology Department (2018R1035-11, 2018J01855, 2019J01338), the Open Research Fund of Fujian Key Laboratory of Natural Medicine Pharmacology, Fujian Medical University (FJNMP-201801), and the project of Fujian Province Office of Education (JZ160442), the research project of Fujian Provincial Health and Family Planning Commission (2017-1-80).

## Conflict of Interest

The authors declare that the research was conducted in the absence of any commercial or financial relationships that could be construed as a potential conflict of interest.

## Abbreviations

CCA, common carotid artery; Cpu, striatal caudate putamen; DA, dopamine; DOPAC, 3,4-dihydroxyphenylacetic acid; ECA, external carotid artery; ELISA, enzyme-linked immunosorbent assay; Gp, globus pallidus; HPLC-ECD, high-performance liquid chromatography coupled with electrochemical detection; HVA, homovanillic acid; ICA, internal carotid artery; MAO, monoamine oxidase; MCAO, middle cerebral artery occlusion; mNSS, modified neurological severity score; OD, optical density; SNpc, substantia nigra pars compacta; SNpr, substantia nigra pars reticulata; rt-PA, recombinant tissue plasminogen activator; SN, substantia nigra; TH, tyrosine hydroxylase.
